# Supplementary material for: Change in address in electronic health records as an early marker of homelessness
Source: PLoS One. 2025 Mar 10;20(3):e0318552. doi: 10.1371/journal.pone.0318552 (PMC11892818; doi:10.1371/journal.pone.0318552)
Supplement: S1 Table — (DOCX) [file pone.0318552.s001.docx]

Supplement

**S1 Table. Top ten diagnoses and prevalence per address churn group.**

| Address Churn Group | Diagnosis | Raw Count | Proportion |
| --- | --- | --- | --- |
| 0 | Essential (primary) hypertension | 231,317 | 0.306 |
| 0 | Vitamin D deficiency unspecified | 207,583 | 0.275 |
| 0 | Hyperlipidemia unspecified | 169,842 | 0.225 |
| 0 | Weakness | 158,370 | 0.209 |
| 0 | Pain in unspecified limb | 147,159 | 0.195 |
| 0 | Cough | 142,575 | 0.189 |
| 0 | Pure hypercholesterolemia | 127,880 | 0.169 |
| 0 | Anxiety and fear-related disorders | 120,861 | 0.160 |
| 0 | Other general symptoms and signs | 117,574 | 0.155 |
| 0 | Other abnormal glucose | 117,468 | 0.155 |
| 1 | Vitamin D deficiency unspecified | 43,415 | 0.277 |
| 1 | Essential (primary) hypertension | 42,933 | 0.274 |
| 1 | Pain in unspecified limb | 36,969 | 0.236 |
| 1 | Weakness | 35,655 | 0.227 |
| 1 | Cough | 34,603 | 0.221 |
| 1 | Anxiety and fear-related disorders | 33,820 | 0.216 |
| 1 | Other general symptoms and signs | 30,846 | 0.197 |
| 1 | Hyperlipidemia unspecified | 30,676 | 0.195 |
| 1 | Acute upper respiratory infection unspecified | 30,545 | 0.195 |
| 1 | Low back pain | 29,155 | 0.186 |
| 2 | Vitamin D deficiency unspecified | 39,079 | 0.284 |
| 2 | Anxiety and fear-related disorders | 38,814 | 0.282 |
| 2 | Pain in unspecified limb | 36,443 | 0.265 |
| 2 | Weakness | 34,312 | 0.250 |
| 2 | Essential (primary) hypertension | 33,786 | 0.246 |
| 2 | Cough | 33,106 | 0.241 |
| 2 | Other general symptoms and signs | 33,065 | 0.240 |
| 2 | Acute upper respiratory infection unspecified | 31,856 | 0.232 |
| 2 | Other chronic pain | 29,309 | 0.213 |
| 2 | Low back pain | 29,188 | 0.212 |
| 3 | Anxiety and fear-related disorders | 3,641 | 0.381 |
| 3 | Vitamin D deficiency unspecified | 3,085 | 0.323 |
| 3 | Pain in unspecified limb | 2,978 | 0.312 |
| 3 | Other general symptoms and signs | 2,971 | 0.311 |
| 3 | Weakness | 2,795 | 0.292 |
| 3 | Acute upper respiratory infection unspecified | 2,699 | 0.282 |
| 3 | Anhedonia | 2,673 | 0.280 |
| 3 | Cough | 2,667 | 0.279 |
| 3 | Acute pharyngitis unspecified | 2,641 | 0.276 |
| 3 | Depressive disorders | 2,606 | 0.273 |
| 4 or more | Anxiety and fear-related disorders | 3,694 | 0.454 |
| 4 or more | Pain in unspecified limb | 2,957 | 0.364 |
| 4 or more | Weakness | 2,826 | 0.348 |
| 4 or more | Other general symptoms and signs | 2,824 | 0.347 |
| 4 or more | Vitamin D deficiency unspecified | 2,822 | 0.347 |
| 4 or more | Depressive disorders | 2,649 | 0.326 |
| 4 or more | Cough | 2,631 | 0.324 |
| 4 or more | Other chronic pain | 2,599 | 0.320 |
| 4 or more | Anhedonia | 2,546 | 0.313 |
| 4 or more | Acute pharyngitis unspecified | 2,538 | 0.312 |
