# Supplementary material for: Change in address in electronic health records as an early marker of homelessness
Source: PLoS One. 2025 Mar 10;20(3):e0318552. doi: 10.1371/journal.pone.0318552 (PMC11892818; doi:10.1371/journal.pone.0318552)
Supplement: Attachment — Submitted filename: Response to reviewers.docx [file pone.0318552.s002.docx]

Dear Editors, Reviewers, and Journal Staff,

We sincerely appreciate the time and effort invested in reviewing our manuscript and the constructive feedback provided. In response, we have revised the manuscript and included both a tracked-changes version and a clean version without tracked changes for clarity. Additionally, we have provided an updated cover letter that summarizes the revisions made to the manuscript. A detailed point-by-point response to each of the reviewers’ comments can be found below.

Thank you for your consideration of our manuscript.

Warm regards,

Janet Song, MD

**Response to reviewers**

**1. Inadequate Introduction**

**Reviewer Comment:** Introduction should include proper background of the research area and research topic as well as the works from state of the arts.

**Response:** Thank you for your feedback. We have revised the introduction to provide a more comprehensive background of the research area and topic. Specifically, we provided a broader historical context for our research topic and incorporated state of the art of general homelessness research in lines 55-58 and lines 62-72, respectively. Please see the related works section to get a more comprehensive literature review of housing screening in electronic health records.

**2. Graphics and Visual Aids**

**Reviewer Comment:** All of the figures and tables in the manuscript could be enhanced for clarity and better understanding.

**Response:** We appreciate your suggestion. We have reviewed our figures and processed them through PACE to best adhere to PLOSOne publication standards. We also encourage you to click the links in the PDFs as the figures in the PDF have lower resolution than the actual image accessed through the link. Tables have been reviewed to ensure numbers are clear and accurate.

**3. Formatting and Structure**

**Reviewer Comment:** There are many inconsistencies in the formatting and structure of the manuscript that could be streamlined for a more professional presentation. Authors uses the same heading two times. “Diagnosis data” line 224 and “Diagnoses” in line 307.

**Response:** Thank you for bringing this point of confusion to our attention. We have thoroughly reviewed and corrected the formatting and structure of the manuscript. The headings have been adjusted so the reader may better understand the content contained within each header. In line 203 we have changed “Diagnosis data” to be “Processing diagnosis data” to clarify that we are discussing our methodology of how we processed the diagnosis data. In line 262, we have changed the header from “Diagnoses” to “Diagnostic characteristics of address churn” to clarify that these are the results of our analyses looking at proportions of diagnoses per address churn group as displayed in Figure 2 and looking at top ten most frequent diagnoses per group. This header now parallels the preceding header in line 246, “Demographic characteristics of churn”.

We have additionally included headers in the discussion section to help guide readers (lines 310, 323, 344, 366, 385, and 394). We also restructured the discussion section to better convey our main findings how they support our hypothesis. With the exception of the “Association between address churn and homelessness” section, we have also reordered the discussion section to better parallel the order of the results section in lines 324-344.

We hope these changes help with making the overall presentation streamlined for a more professional appearance.

**4. Related Works Section Missing**

**Reviewer Comment:** Related works section missing.

**Response:** Thank you for your feedback, while there is not a separate related works section, we have a detailed review of related works already included in the introduction. We now include a heading in our paper highlighting the “Related works” section to improve the clarity. The introduction has been edited as discussed in item 1 “Inadequate Introduction” so that it includes a broader overview on the research matter and the relevant related works on housing screening is in a separate “Related works” section on lines 80-107.

**5. Overall Presentation**

**Reviewer Comment:** In overall, this manuscript looks like a technical web report.

**Response:** We appreciate your candid feedback. We have made significant revisions as detailed above to ensure that the manuscript adheres to academic standards and does not resemble a technical web report. These changes include improving the narrative flow, enhancing the academic tone, and ensuring consistency in formatting and style.
