# Supplementary material for: Change in address in electronic health records as an early marker of homelessness
Source: PLoS One. 2025 Mar 10;20(3):e0318552. doi: 10.1371/journal.pone.0318552 (PMC11892818; doi:10.1371/journal.pone.0318552)
Supplement: Attachment — Submitted filename: pone.0318552.docx [file pone.0318552.s003.docx]

Change in address in electronic health records as an early marker of homelessness

Janet Song ^1,2 *^, Yu Deng ^1,2,3^, Yuyang Yang ^1,2^, Lacey Gleason ^1,2^, Abel Kho ^1,2,4^

^1^ Feinberg School of Medicine, Northwestern University, Chicago, IL, United States of America.

^2^ Center for Health Information Partnerships, Northwestern University Feinberg School of Medicine, Chicago, IL, United States of America.

^3^ AbbVie Inc. Chicago, IL, United States of America.

^4^Division of General Internal Medicine, Department of Medicine, Feinberg School of Medicine, Northwestern University, Chicago, IL, United States of America.

***** Corresponding Author:

Email: [Janet.song80@gmail.com](mailto:Janet.song80@gmail.com) (JS)

**Abstract**

**Introduction**

Housing stability is a key health determinant and there is a need for early screening for instability with existing electronic health record (EHR) data to improve health outcomes. We aim to establish recorded address changes as a screening variable for housing instability and homelessness and to attempt to define the threshold of high churn.

**Methods**

Our study is a single-center cross-sectional study of EHR data (2018-2024) conducted at a US academic center with eleven sites across Chicago. We include patients 18 years or older with at least three hospital encounters over three different years. We define address churn as the number of address changes recorded in the EHR corrected to three-year intervals. We compare demographic and clinical characteristics of individuals with varying address churn with the student T-test to look at distribution of address churn for patients with and without record of homelessness, ANOVA to evaluate the distribution of ages for different levels of churn, and the chi-square test to evaluate for association between churn and clinical diagnoses. We perform multivariable logistic regression to measure the association between people with a record of homelessness and address changes.

**Results**

The study includes 1,068,311 patients with 756,222 having zero address changes, 156,911 having one address change, 137,491 with two address changes, 9,558 with three address changes, and 8,129 with four or more address changes. People with no record of homelessness in the EHR have mean address changes of 0.6 (SD 0.7) whereas people with record of homelessness have mean address changes of 1.8 (SD 1.3). Diagnostic profiles of the varying address change groups show increased prevalence of psychiatric diagnoses (65.2% in the 4 or more-address change group) compared to lower address change (27.7% in the 0-address change group). Address churn is significantly associated with homelessness with an odds ratio (OR) of 1.44 (95% CI = [1.42-1.47], P <0.001).

**Conclusion**
Our results support a role for residential address churn in screening for housing instability in healthcare systems and reinforce the association between psychiatric disorders and housing instability. Our findings can help public health policy makers in targeting vulnerable populations at risk of homelessness with multiple health comorbidities for housing interventions.

**Introduction**

Homelessness was estimated to affect 582,462 people in the United States in 2022, disproportionately impacting individuals identifying as Black or African American [1]. Historically, homelessness has been influenced by economic downturns, housing policies, and social safety nets, with significant spikes during periods such as the Great Depression and the post-industrial era [2]. Recent trends indicate a rise in chronic homelessness, exacerbated by the COVID-19 pandemic [3].

Some consider homelessness as one extreme of the housing stability spectrum with stable housing on the other end [4]. Housing instability encompasses a range of challenges including trouble paying rent, overcrowding, frequent residential moves, or spending a large portion of one’s income on housing. These issues are intertwined with broader social determinants of health, such as education, employment, and social support networks [5].

The landscape of homelessness research has evolved, highlighting the critical need for evidence-based policy responses. Initiatives such as Housing First and supportive housing programs have emerged as effective strategies to address chronic homelessness by offering stable housing without preconditions [6–8]. However, the implementation of these programs often faces significant barriers, including funding limitations, policy inconsistencies, and systemic challenges that hinder effective outreach and support for the homeless population [9–11].

Homelessness and housing instability greatly impact health and healthcare costs. They have been linked with increased psychiatric disorders, substance abuse, cardiovascular disorders, infectious disease, and all-cause mortality [12–15]. Homelessness is also associated with increased emergency department (ED) utilization and longer hospital stays [16,17]. While research is ongoing, the link between housing and health appears multifaceted and intersects with issues such as accessibility and social support [18]. Effective screening for housing instability can help reduce incidence of unmanaged disease, decrease healthcare costs, and uphold patient-centered care [19].

**Related works**

Current methods using EHR based screening tend to miss patients experiencing homelessness [20]. The International Statistical Classification of Diseases and Related Health Problems: 10th Revision (ICD-10), Z-codes were first made available in the 2016 fiscal year to track social determinants of health in patient records [21]. However, usage of these codes has been infrequent, as low as 2.03% of patients with Z-codes recorded [22]. Other strategies include adding structured fields to capture social determinants of health (SDOH) but similarly showed low utilization with less than 1% of encounters having structured fields completed [23]. While providers often recognize the importance of addressing SDOH, they report multiple barriers in recording or retrieving the psychosocial history such as discomfort discussing sensitive topics with patients, limited time in clinic, and logistical difficulties retrieving data from EHR [24,25].

Screening questionnaires tend to be more time intensive and miss a significant portion of homeless individuals [26]. Integrating screening with intervention has shown great potential and praise from patients but often is difficult for providers and resource intensive for health systems [27,28]. As a result, follow-up for patients who are identified is often poor [29,30].

Natural language processing (NLP) in EHRs has become an increasingly popular method to scan notes to identify patients experiencing homelessness but has yet to make its way into real-world practice [31–33]. NLP model accuracy is only as good as how well SDOH are documented in the EHR and is often not generalizable as the training occurs with a specific health system and patient population [34–36]. Furthermore, these methods identify patients who are already homeless, not those at risk or on the verge of homelessness. Developing a social risk score is promising as it would use data that does not rely on providers such as Z-codes and would not suffer in accuracy depending on free text quality as in NLP, but current performance is not yet acceptable as there is a need to explore more variables to create a more robust score [37]. Our study attempts to fill in the gaps in SDOH screening in health systems by exploring frequency of address changes, an unbiased variable that is readily available and regularly recorded at the time of intake.

While a variety of screening tools and methods exist using electronic health record data (EHR), housing stability remains difficult to track. We hypothesize that frequency of address changes, or address churn, is a marker of housing instability and is associated with poor health outcomes. We evaluate our hypothesis by running a multivariate logistic regression analysis with data available in the EHR to test if there is an association between address changes and homelessness. Given that address changes are poorly described in the literature, our study also seeks to explore how diagnoses vary with different levels of churn. We answer this question by performing a chi-square analysis for diagnoses and address churn. We also explore how demographic characteristics vary with address churn. We answer this question using mixed methods, combining statistical subgroup analyses and qualitative geospatial analysis. This comprehensive analysis of address churn can help fill in the gaps for patients who are not asked about their living situation or whose narrative is obscured in the EHR.

**Methods**

**Data and study population**

We utilize a retrospective cross-sectional study design to get a baseline snapshot of address churn using Northwestern Medicine’s (NM) Electronic Data Warehouse (EDW) EHR data including the dates 1/1/2018-1/1/2024. The NM-EDW is a data repository that includes clinical and research data from sites within Northwestern University School of Medicine and Northwestern Medicine Healthcare (NMHC) for greater than 10 million patients spanning from 1970 to the present. It contains demographic data (age, gender, race, address), clinical data (diagnoses, medications, encounters, procedures), and outcomes extracted from the Northwestern Medicine EHR. The data was accessed for research on 3/5/2024. Authors had access to MRNs, which could identify participants, after data collection. We include adult patients who were at least 18 years of age as of 1/1/2018. To better capture residential address mobility over time, we include patients with three or more encounters spread across three different years. This results in 1,068,311 patients included in our study. We use structured data from the EHR including diagnosis recorded as ICD-10 codes, residential address, insurance provider, age, gender, and race. Gender and race are self-reported and extracted as recorded in the EHR. Data on race was collected due to the complex nature of race and housing [38]. The race group “Other” in this study includes patients who declined to report race or recorded race of “unknown”. The study protocol was approved by the Northwestern University Institutional Review Board. Waiver of informed consent was obtained for this study because the research involved no more than minimal risk to patients as this study uses pre-existing data, the waiver will not adversely affect the rights and welfare of participants, and it would not be practicable to carry out the research without the waiver.

**Address standardization**

Residential address is input in a free text format at the time of patient intake and may be revised at check-in for follow-up appointments and admissions. Date and time of the residential address record are included as metadata. The free text nature of the residential address results in spelling variations which creates an additional address record. We process residential addresses using the United States Postal Service (USPS) Postal Addressing Standards. The USPS Postal Addressing Standards is a comprehensive set of guidelines that ensure proper address formatting for efficient mail processing and delivery [39]. We first convert all address text to uppercase. We create dictionaries of different abbreviations and common spelling errors for street suffix (i.e. Ave, Avenue, Av, Avn), directional indicators (north, south, east, west), and state names mapped to the standard USPS abbreviation. Standardized formatting for address spelling and capitalization allows us to decrease overcounting of address changes for more accurate calculation of address churn as described below.

**Identifying individuals who experienced homelessness**

We consulted with housing workgroup experts to identify patients who have experienced homelessness. With the housing workgroup’s guidance, we developed the following initial criteria to find patients experiencing homelessness which included ICD-10 code of Z59.0, residential address that either indicates “undomiciled” or “homeless”, congregate living facility as home address, or hospital address as home address [32,40].

We conducted a blinded chart review of 50 randomly selected patients, stratified by housing status with 10 that met criteria for homelessness and 40 patients without criteria for homelessness. Among the 40 patients who did not meet the criteria for homelessness, we found no evidence of any previous experiences of homelessness in their medical charts. Of the 10 patients who met the criteria for homelessness, only 5 had documented evidence of homelessness in their charts. The remaining 5 patients, despite meeting the criteria for homelessness, did not have documentation of homelessness in their charts. Instead, these 5 patients were identified as homeless based on their use of the hospital address. Based on the results from our chart review, we were initially overcounting homelessness by including patients who have the hospital as their address. We remove this criterion and conduct our study based on the final criteria: ICD-10 code of Z59.0, residential address that either indicates “undomiciled” or “homeless”, and congregate living facility as home address.

Table 1 provides a summary of patients included in our study with approximately 0.2% of our study population having record of homelessness.

**Table 1. Baseline demographic variables.**

| Group | 0 Address Churn (N=756,222) | 1 Address Churn (N=156,911) | 2 Address Churn (N=137,491) | 3 Address Churn (N=9,558) | 4 or More Address Churn (N=8,129) |
| --- | --- | --- | --- | --- | --- |
| Age Mean (SD) | 52.1 (16.5) | 46.2 (17.2) | 41.9 (16.9) | 38.1 (15.9) | 37.9 (16.0) |
| Sex |  |  |  |  |  |
| Female Count (%of group) | 472,782 (62.5%) | 101,446 (64.7%) | 92,164 (67.0%) | 6,784 (71.0%) | 5,957 (73.3%) |
| Male Count (% of group) | 283,440 (37.5%) | 55,465 (35.3%) | 45,327 (33.0%) | 2,774 (29.0%) | 2,172 (26.7%) |
| Race (% of group) |  |  |  |  |  |
| White | 505,766 (66.9%) | 107,414 (68.5%) | 95,711 (69.6%) | 6,663 (69.7%) | 5,656 (69.6%) |
| Black or African American | 39,420 (5.2%) | 13,341 (8.5%) | 14,617 (10.6%) | 1,293 (13.5%) | 1,223 (15.0%) |
| Asian | 24,179 (3.2%) | 6,809 (4.3%) | 6,375 (4.6%) | 398 (4.2%) | 315 (3.9%) |
| AI/AN/NHPI | 1,622 (0.2%) | 463 (0.3%) | 450 (0.3%) | 30 (0.3%) | 38 (0.5%) |
| Other | 185,235 (24.5%) | 28,884 (18.4%) | 20,338 (14.8%) | 1,174 (12.3%) | 897 (11.0%) |
| Insurance (% of group) |  |  |  |  |  |
| Commercial | 402,656 (53.2%) | 93,449 (59.6%) | 88,421 (64.3%) | 6,464 (67.6%) | 5,187 (63.8%) |
| Medicaid | 35,402 (4.7%) | 11,491 (7.3%) | 12,227 (8.9%) | 1,048 (11.0%) | 1,119 (13.8%) |
| Medicare | 233,307 (30.9%) | 37,248 (23.7%) | 25,742 (18.7%) | 1,440 (15.1%) | 1,375 (16.9%) |
| Other | 84,857 (11.2%) | 14,723 (9.4%) | 11,101 (8.1%) | 606 (6.3%) | 448 (5.5%) |
| ADI Mean (SD) | 0.30 (0.09) | 0.31 (0.09) | 0.31 (0.08) | 0.32 (0.08) | 0.32 (0.08) |
| Record of Homelessness (% of group) |  |  |  |  |  |
| No record | 755,780 (99.9%) | 156,567 (99.8%) | 136,660 (99.4%) | 9,440 (98.8%) | 7,847 (96.5%) |
| Positive Record | 442 (0.1%) | 344 (0.2%) | 831 (0.6%) | 118 (1.2%) | 282 (3.5%) |

AI/AN/NHPI = American Indian/Alaskan Native/Native Hawaiian and Pacific Islander

**Characterizing and defining address churn**

We define address churn as the number of times a person’s residential address changes over time. In this study, we quantify address changes corrected to three-year intervals to standardize the data across different time spans of patient encounters. For instance, a patient with encounter data spanning six years with six instances of address changes is adjusted to an average of three address changes per three-year interval. Address churn is calculated by counting each instance the patient’s address record changed. Addresses are standardized as above to decrease overcounting address record changes. Here, we capture the demographic variances for different levels of address churn. We plot the distribution of address churn as a bar plot for people who have record of homelessness in the EHR and people who have no record of homelessness to establish a threshold for high address churn. To understand address churn in the context of Chicago neighborhoods, we map the geospatial distribution of churn. We calculate the mean address churn per zip code, plot it on a map of Chicago from the U.S. Census Bureau, and compare it to a map of area deprivation index (ADI) per zip code [41]. ADI accounts for several measures of SDOH and is normalized to a scale of 0 to 1 with greater values corresponding to greater deprivation and lower socioeconomic standing [42].

Address churn is categorized into groups of 0 address changes, 1 address change, 2 address changes, 3 address changes, and 4 or more address changes to compare prevalence of diagnoses and age and gender distributions.

**Processing diagnosis data**

For the multivariate regression analysis, we code whether a patient has ever had a diagnosis of severe mental illness or substance abuse as these have been shown to be risk factors for experiencing homelessness. We use the Healthcare Cost and Utilization Project’s (HCUP) Level 2 groupings for behavioral health diagnoses to create more meaningful groups and include ICD-10 codes from groups beyond F00-F99 pertaining to behavioral health [15,43].

To address our question of how diagnoses change with varying levels of address churn, we tabulate diagnosis frequencies by churn group and identify the top ten diagnoses. We group diagnoses from the top ten into cardiovascular diagnoses (essential hypertension, hyperlipidemia, and pure hypercholesterolemia), psychiatric diagnoses (anxiety and fear-related disorders, depressive disorder, and anhedonia), other signs and symptoms (other signs and symptoms, other abnormal glucose, vitamin D deficiency, and weakness), infectious symptoms and diagnoses (cough, acute respiratory infection unspecified, and acute pharyngitis unspecified), and pain-related diagnoses (knee pain and other chronic pain). An age-stratified chi-squared test assesses the association between diagnoses and churn levels. Age groups include young adult (18-35 years), adult (35-65 years), and senior (65+ years) to account for any between age group differences in diagnoses.

**Confounding variables**

As we evaluate the association between homelessness and address churn, we account for several confounding variables. Insurance status, which can act as a confounder and a proxy for economic status, is adjusted for by including it in our model [44,45]. Neighborhood factors, which likely influence housing status, are approximated using the ADI and are corrected for by incorporating it into our model. Mental illness, another potential confounder, is controlled for by including it in the model. Further details of the model are provided in the next section.

**Statistical analysis**

To test our hypothesis that there is an association between the history of homelessness (binary outcome) and address churn, we use multivariate logistic regression. Address churn is the predictor variable of interest and is an integer ranging from 0 to 11. We run both univariate and multivariate models, adjusting for relevant demographic and clinical variables including age, self-reported gender and race as recorded in the EHR, insurance status, mean ADI of a patient’s collective addresses, any diagnosis of severe mental illness (bipolar, schizophrenia, or psychosis), and any diagnosis of substance use disorder. We fit a logistic regression model using the statsmodels module in Python. All analyses are completed in Python v3.6.

To answer the question of how patient demographic characteristics varied with different levels of address churn, we utilize various statistical tests. We run a student T-test to compare distributions of address churn for patients with record of homelessness and those without. We evaluate how age and address churn vary with an ANOVA comparing age distributions across different levels of address churn. The statsmodels module is used to run the analyses listed above.

**Results**

**Demographic characteristics of address churn**

We observed significant differences in demographics across levels of address churn. The majority of the study population did not move over three years. Patients with a record of homelessness showed a mean churn of 1.83 (SD 1.34), significantly higher than patients without homelessness records, who had a mean churn of 0.58 (SD 0.71) (Fig 1A). A student T-test demonstrates that the difference is significant (p-value <0.001). The distributions of age and gender differ across the levels of churn. The mean age decreases across the varying churn groups from a mean age of 52 in the zero-churn group to a mean age of 38 in the four or more-churn group (Fig 1B). A one-way ANOVA compares the age distribution across four groups, revealing a statistically significant difference in age distribution between the groups. Post hoc Tukey test reveals that age across groups was significantly different (p < 0.001 for all pairwise comparisons).

**Fig 1. Address churn distributions by homeless status and demographics.** A) Distributions of address churn by people without record of homelessness in the EHR and people with record of homelessness in the EHR. B) Distributions of age and sex per address churn group. Mean age of each group indicated by dotted black line.

**Diagnostic characteristics of address churn**

We find diagnoses differ across the different levels of address churn. A chi-squared analysis looking at the five diagnostic groups and the five churn groups finds diagnosis is significantly associated with churn group for young adults, adults, and seniors. Prevalence of psychiatric diagnoses increases as address churn increases starting with 27.7% of patients in the zero-address churn group compared to 65.2% in the four or more-address churn group. Pain and infectious diagnoses similarly increase across groups while cardiovascular diagnoses decrease (Fig 2).

**Fig 2. Diagnostic profiles of address churn groups.** Proportions of diagnosis groups: other general symptoms and signs, cardiovascular, pain, psychiatric, and infectious per address churn groups.

Looking more granularly at the diagnoses per group, we find hypertension is the most common diagnosis for people with zero address churn with a prevalence of 30.6%, followed by vitamin D deficiency and hyperlipidemia. In the one-address churn group, vitamin D deficiency and hypertension are the top two most common diagnoses (27.7% and 27.4% respectively), with anxiety and fear-related diagnoses ranking sixth at 21.6%. Anxiety and fear-related disorders are the second most common diagnosis in the two-address churn group (28.2%), the most common diagnosis for the three-address churn group (38.1%) and reaching a peak in the four or more-address churn group (45.4%). Anhedonia, depressive disorders, and pain-related disorders are notably frequent in the latter group (S1 Table).

**Logistic regression model**

We find that address churn is significantly associated with ever experiencing homelessness before and after adjustment with other demographic variables with increased odds of 1.44 95% CI [1.42 - 1.47] for each additional residential address change. Male gender (OR 3.43 95% CI [3.09 - 3.79]), EHR recorded self-identified black or African American race (OR 3.42 95% CI [3.03 - 3.84]), insurance status especially Medicaid (OR 22.05 95% CI [18.48 - 26.30]), previous diagnosis of severe mental illness (OR 5.77 95% CI [5.18 - 6.43]), previous diagnosis of substance abuse (OR 4.19 95% CI [3.77 - 4.65]), and deprivation index (OR 1.30 95% CI [1.23 - 1.37]) are also found to be significantly associated with record of experiencing homelessness (Table 2).

**Table 2. Univariate and multivariate logistic regression odd ratio and 95% CI.**

|  | Univariate | | Multivariate | |
| --- | --- | --- | --- | --- |
| Variable | Odds Ratio [95% CI] | p-value | Odds Ratio [95% CI] | p-value |
| Age | 0.99 [0.99 - 1.0] * | <0.001 | 1.00 [0.99 - 1.0] | 0.335 |
| Sex (Reference Female) |  |  |  |  |
| Male | 3.34 [3.04 - 3.66] * | <0.001 | 3.43 [3.09 - 3.79] * | <0.001 |
| Race (Reference White) |  |  |  |  |
| Black or African American | 10.64 [9.71 - 11.66] * | <0.001 | 3.42 [3.03 - 3.84] * | <0.001 |
| Asian | 0.70 [0.5 - 0.98] * | 0.040 | 1.08 [0.76 - 1.53] | 0.679 |
| AI/AN/NHPI | 2.11 [1.0 - 4.44] | 0.050 | 1.68 [0.77 - 3.64] | 0.19 |
| Other | 0.36 [0.3 - 0.44] * | <0.001 | 0.51 [0.41 - 0.63] * | <0.001 |
| Churn | 1.66 [1.64 - 1.69] * | <0.001 | 1.44 [1.42 - 1.47] * | <0.001 |
| Insurance (Reference Commercial Insurance) |  |  |  |  |
| Medicaid | 73.16 [61.85 - 86.55] * | <0.001 | 22.05 [18.48 - 26.30] * | <0.001 |
| Medicare | 7.63 [6.4 - 9.11] * | <0.001 | 6.03 [4.95 - 7.36] * | <0.001 |
| Other | 4.00 [3.14 - 5.09] * | <0.001 | 5.66 [4.43 - 7.22] * | <0.001 |
| Area Deprivation Index | 1.92 [1.85 - 1.99] | 0.069 | 1.30 [1.23 - 1.37] * | <0.001 |
| Severe Mental Illness | 29.94 [27.36 - 32.77] * | <0.001 | 5.77 [5.18 - 6.43] * | <0.001 |
| Substance Abuse Diagnosis | 19.53 [17.87 - 21.36] * | <0.001 | 4.19 [3.77 - 4.65] * | <0.001 |

CI = Confidence interval.

AI/AN/NHPI = American Indian/Alaskan Native/Native Hawaiian and Pacific Islander

* = P-value <0.05 considered significant.

**Geospatial analysis of churn and ADI**

The plot of mean patient address churn per zip code shows that the areas in central Chicago tend to have patients residing with higher mean address churn, with River North (zip code 60654) having the highest average patient address churn of 1.55 but low ADI 0.26 On the other hand, areas in southwest and west Chicago have the highest ADI. In particular, the neighborhoods of Englewood and Washington Park (zip code 60621) in the southwest region of Chicago have ADI of 0.69 and address churn of 1.17 and South Lawndale (zip code 60623) in the west has ADI of 0.68 and address churn of 0.87 (Fig 3A and B).

**Fig 3. Geospatial distributions of address churn and ADI in Chicago.** A) Average address churn per zip code in Chicago. B) ADI per zip code in Chicago.

**Discussion**

**Overview**

In our study we find that address churn is significantly associated with having experienced homelessness with increased odds of 1.44 [95% CI 1.42 - 1.47]. Other variables associated with ever experiencing homelessness include self-reported race, with EHR recorded race as Black or African American compared to recorded race of White having the greatest odds ratio of 3.42 (95% CI [3.03 - 3.84]), insurance status with Medicaid compared to commercial insurance having greatest odds ratio of 22.05 (95% CI [18.48 - 26.3]), diagnosis of SMI, diagnosis of substance abuse, and area deprivation index. We observe demographic variability among the different address churn groups with regards to age, housing status, and neighborhood. We also show diagnostic differences across the levels of address churn with greater address churn tending towards greater frequency of anxiety, mood-related diagnoses, and chronic pain, while lower churn groups tend to have higher frequency of “diseases of affluence” in the zero-address churn group.

**Association between address churn and homelessness**

Our finding that address churn is statistically significantly associated with homelessness supports our hypothesis that it is a marker for housing instability, as housing status exists on a continuum with the most severe instability being homelessness [4,46]. To our knowledge our study is the first to explore address churn in the EHR as a measure of housing instability [22,27,28,36,37]. We propose that address churn, a variable that is already recorded in EHRs, can contribute to improved screening for individuals experiencing housing instability or individuals at risk of homelessness. Address churn shows a significant effect in the univariate and multivariate models, but the effect size is attenuated in the full model, suggesting that, although churn is an important predictor on its own, its contribution is moderated by other SDOH. Existing literature similarly supports the idea that a multitude of external factors including mental health, income, and neighborhood safety may play a role in homelessness [47–49]. As these processes occur, an individual may experience unstable housing requiring frequent address changes [50]. Recognizing housing instability is not just relevant for housing interventions [51,52]. It is also a public health issue as it has been shown to exacerbate chronic conditions like heart failure, hypertension, and COPD, heighten the risk of infectious diseases and violence, and incur higher healthcare costs due to longer hospital stays and more emergency visits [12–14,16,53–57]. Understanding address churn in the context of other demographic factors can improve screening in clinics and contribute to improving health outcomes associated with homelessness and housing instability.

**Describing and characterizing address churn**

As we attempt to define a threshold for high churn, we find there is no consensus for what it means to have high residential mobility. The U.S. Census finds that on average, Americans move 11.7 times in their lifetimes, while other studies find that three or four residential moves regardless of timespan may be considered high mobility [58,59]. Our findings add to the literature by taking time interval into consideration. We observe patients who have no record of homelessness have average address churn of less than one in a three-year period, while patients who have history homelessness have an average churn of about two in a three-year interval.

To better characterize churn, we observe demographic differences with varying levels of churn. Most patients included in the study do not move over three years. This indicates a general stability in housing for much of the patient population. Patients with a record of homelessness have a statistically significant higher mean churn compared to those without, highlighting the instability faced by individuals with a history of homelessness. The higher address churn among these patients suggests ongoing housing insecurity, which further supports our main hypothesis that address churn is an early marker of homelessness. A one-way ANOVA reveals a statistically significant difference in age distribution between the address churn groups. Younger individuals are more likely to experience higher address churn compared to older individuals. This may be due to various factors such as employment instability, educational pursuits, or lack of established housing. Previous literature also supports the idea of “ageing-in-place” for older adults in metropolitan areas and that moves are generally motivated by major life events [60,61].

**Diagnosis and association with churn**

Each group of address churn shows a distinct diagnostic profile. Address churn and diagnosis, stratified by age, are significantly associated. Meaning, while young adults have greater prevalence of mood disorders compared to older adults this does not fully explain the differences in diagnoses [62]. We observe that people in higher address churn groups have greater frequencies of psychiatric diagnoses, specifically, anxiety and fear-related disorders. This may be due to the stressors associated with residential mobility which is often reactive to situations like evictions and living conditions [63–66]. This finding highlights the potential mental health risks associated with high address churn and underscores the need for targeted mental health support for these populations. The higher frequency of pain-related disorders in the higher churn groups may be attributed to pain being more poorly controlled, exacerbated by lack of consistent access to healthcare and poor living conditions [67,68]. This finding emphasizes the importance of ensuring continuous healthcare access and support for individuals with high address churn. Overall, the common diagnoses in the high churn groups are like diagnoses seen in people experiencing homelessness [15,69–71]. The lower churn groups have greater prevalence of diseases such as hypertension and hyperlipidemia, often affecting older and wealthier individuals. Alternatively, it could indicate underdiagnosis or lack of access to healthcare services among those with high address churn.

**Geospatial patterns of churn**

Areas of high churn do not necessarily overlap with areas of high deprivation, which may be explained by the patterns of immobility seen in areas of lower socioeconomic status. Reports show that generally families in lower socioeconomic standing report living in the same residence until an external factor like extreme violence forces them to move elsewhere [63,72]. River North, a downtown Chicago neighborhood with high housing costs, has the highest address churn and corresponds to areas of higher homelessness per the Chicago Point-in-Time estimate and may be driving up the average churn of the area [73].

**Limitations**

Our study has several limitations and may be subject to several potential biases. Measurement bias could arise from inaccuracies in recording address changes or diagnoses in the medical records. Measurement bias may also arise as there is no standardized method of identifying homelessness. Our method of using address and ICD-10 codes relies on the clinician’s screening ability and the patient’s honesty. As a result, ours is likely a conservative estimate of the number of individuals experiencing homelessness in the EHR. Information bias may occur as the NM-EDW inherently possesses limitations that may affect the validity of research findings including issues related to documentation errors and missing data. We attempt to mitigate these biases by standardizing addresses prior to analysis and by validating our data through chart review. The generalizability of our findings may be limited by the specific characteristics of our study population. Our study took place during the COVID-19 pandemic which may skew results due to the volatility of housing and healthcare usage during this time [74,75]. We attempt to temper the effects of COVID-19 by including three additional years (2021-2024) to improve generalizability of our findings. The population included in our study may not be fully representative of Chicago as we pulled patients from the Northwestern health system, which has an overlapping catchment area with other large health systems. The population is primarily patients from an urban healthcare system which may not be generalizable to rural settings. Future research should aim to replicate our study in diverse settings to enhance the generalizability of the results.

Several assumptions were made during our analysis. While this study simplifies cases of homelessness to binary categorization, we acknowledge that a broad range of housing instability and homelessness exist, and more granular exploration may be necessary but are beyond the scope of this paper. We recognize that housing is complex and residential address churn may be one dimension among many [76]. We acknowledge that residential mobility can reflect positive change (i.e. moving towards more stable housing) but literature supports that it is more frequently a symptom of housing instability [77–79].

**Conclusion**

In conclusion, our study finds a statistically significant association between address churn and homelessness, which highlights a potential avenue for administrative screening to detect signs of housing instability with variables already routinely captured in EHRs. Traditional indicators including ICD-10 codes and address records may not be as effective at identifying the nuances in housing instability or in identifying individuals at risk before they experience homelessness. We find higher prevalence of anxiety, mood disorders, and chronic pain among those with higher churn, indicating a link between psychosocial stressors and health. While most patients have a churn of zero, we find that people with record of homelessness and young adults tend to have higher churn. Knowing who and where high address churn occurs can help create targeted screening and policy changes. Standardized screening protocols can be supported through collaboration between healthcare providers, social services, and housing agencies. Policymakers should advocate for stable housing as a health determinant and allocate resources to housing stability initiatives. Our findings underscore a potential measure to facilitate the integration of housing stability screening into healthcare systems to identify at-risk individuals early and provide timely interventions.

**Acknowledgments**

This work was supported in part by the Northwestern Medicine Enterprise Data Warehouse*.*

**References**

1.	The U.S. Department of Housing and Urban Development (HUD). The 2022 Annual Homelessness Assessment Report (AHAR) to Congress. 2022.

2.	Meyer Angela Wyse Gillian Meyer Alexa Grunwaldt Derek Wu BD, Abowd J, Asiala M, Carter G, Christy J, Culhane D, et al. Homelessness and the Persistence of Deprivation: Income, Employment, and Safety Net Participation. 2024 [cited 12 Oct 2024]. doi:10.3386/W32323

3.	Leifheit KM, Chaisson LH, Medina JA, Wahbi RN, Shover CL. Elevated Mortality Among People Experiencing Homelessness With COVID-19. Open Forum Infect Dis. 2021;8. doi:10.1093/OFID/OFAB301

4.	Frederick TJ, Chwalek M, Hughes J, Karabanow J, Kidd S. HOW STABLE IS STABLE? DEFINING AND MEASURING HOUSING STABILITY. J Community Psychol. 2014;42: 964–979. doi:10.1002/JCOP.21665

5.	Tsai J, Szymkowiak D, Jutkowitz E. Developing an operational definition of housing instability and homelessness in Veterans Health Administration’s medical records. PLoS One. 2022;17. doi:10.1371/JOURNAL.PONE.0279973

6.	Latimer EA, Rabouin D, Cao Z, Ly A, Powell G, Aubry T, et al. Costs of services for homeless people with mental illness in 5 Canadian cities: a large prospective follow-up study. CMAJ Open. 2017;5: E576–E585. doi:10.9778/CMAJO.20170018

7.	Peng Y, Hahn RA, Finnie RKC, Cobb J, Williams SP, Fielding JE, et al. Permanent Supportive Housing with Housing First to Reduce Homelessness and Promote Health among Homeless Populations with Disability: A Community Guide Systematic Review. J Public Health Manag Pract. 2020;26: 404. doi:10.1097/PHH.0000000000001219

8.	Gouse I, Walters S, Miller-Archie S, Singh T, Lim S. Evaluation of New York/New York III permanent supportive housing program. Eval Program Plann. 2023;97. doi:10.1016/J.EVALPROGPLAN.2023.102245

9.	Kerman N, Kidd SA, Mutschler C, Sylvestre J, Henwood BF, Oudshoorn A, et al. Managing high-risk behaviours and challenges to prevent housing loss in permanent supportive housing: a rapid review. Harm Reduct J. 2023;20: 1–18. doi:10.1186/S12954-023-00873-Z/TABLES/5

10.	Latimer EA, Rabouin D, Cao Z, Ly A, Powell G, Adair CE, et al. Cost-effectiveness of Housing First Intervention With Intensive Case Management Compared With Treatment as Usual for Homeless Adults With Mental Illness: Secondary Analysis of a Randomized Clinical Trial. JAMA Netw Open. 2019;2: e199782–e199782. doi:10.1001/JAMANETWORKOPEN.2019.9782

11.	Stergiopoulos V, Gozdzik A, Misir V, Skosireva A, Connelly J, Sarang A, et al. Effectiveness of Housing First with Intensive Case Management in an Ethnically Diverse Sample of Homeless Adults with Mental Illness: A Randomized Controlled Trial. PLoS One. 2015;10: e0130281. doi:10.1371/JOURNAL.PONE.0130281

12.	Jayawardana S, Mossialos E. Lives cut short: socioeconomic inequities, homelessness, and cardiovascular disease. Eur Heart J. 2020;41: 4021–4023. doi:10.1093/EURHEARTJ/EHAA796

13.	Nanjo A, Evans H, Direk K, Hayward AC, Story A, Banerjee A. Prevalence, incidence, and outcomes across cardiovascular diseases in homeless individuals using national linked electronic health records. Eur Heart J. 2020;41: 4011–4020. doi:10.1093/EURHEARTJ/EHAA795

14.	Snyder LD, Eisner MD. Obstructive lung disease among the urban homeless. Chest. 2004;125: 1719–1725. doi:10.1378/CHEST.125.5.1719

15.	Trick WE, Rachman F, Hinami K, Hill JC, Conover C, Diep L, et al. Variability in comorbidites and health services use across homeless typologies: multicenter data linkage between healthcare and homeless systems. BMC Public Health. 2021;21: 1–9. doi:10.1186/S12889-021-10958-8/TABLES/3

16.	Thakarar K, Morgan JR, Gaeta JM, Hohl C, Drainoni ML. Predictors of Frequent Emergency Room Visits among a Homeless Population. PLoS One. 2015;10. doi:10.1371/JOURNAL.PONE.0124552

17.	Kushel MB, Gupta R, Gee L, Haas JS. Housing instability and food insecurity as barriers to health care among low-income Americans. J Gen Intern Med. 2006;21: 71–77. doi:10.1111/J.1525-1497.2005.00278.X

18.	Kirby JB, Kaneda T. Access to health care: does neighborhood residential instability matter? J Health Soc Behav. 2006;47: 142–155. doi:10.1177/002214650604700204

19.	Chhabra M, Sorrentino AE, Cusack M, Dichter ME, Montgomery AE, True G. Screening for Housing Instability: Providers’ Reflections on Addressing a Social Determinant of Health. J Gen Intern Med. 2019;34: 1213. doi:10.1007/S11606-019-04895-X

20.	Harle CA, Wu W, Vest JR. Accuracy of Electronic Health Record Food Insecurity, Housing Instability, and Financial Strain Screening in Adult Primary Care. JAMA. 2023;329: 423–424. doi:10.1001/JAMA.2022.23631

21.	Medicine I of. Capturing Social and Behavioral Domains and Measures in Electronic Health Records: Phase 2. Capturing Social and Behavioral Domains and Measures in Electronic Health Records: Phase 2. 2014; 1–351. doi:10.17226/18951

22.	Guo Y, Chen Z, Xu K, George TJ, Wu Y, Hogan W, et al. International Classification of Diseases, Tenth Revision, Clinical Modification social determinants of health codes are poorly used in electronic health records. Medicine (United States). 2020;99. doi:10.1097/MD.0000000000023818

23.	Wang M, Pantell MS, Gottlieb LM, Adler-Milstein J. Documentation and review of social determinants of health data in the EHR: measures and associated insights. Journal of the American Medical Informatics Association. 2021;28: 2608–2616. doi:10.1093/JAMIA/OCAB194

24.	Senteio C, Veinot T, Adler-Milstein J, Richardson C. Physicians’ perceptions of the impact of the EHR on the collection and retrieval of psychosocial information in outpatient diabetes care. Int J Med Inform. 2018;113: 9–16. doi:10.1016/J.IJMEDINF.2018.02.003

25.	Kepper MM, Walsh-Bailey C, Prusaczyk B, Zhao M, Herrick C, Foraker R. The adoption of social determinants of health documentation in clinical settings. Health Serv Res. 2023;58: 67–77. doi:10.1111/1475-6773.14039

26.	Cusack M, Montgomery AE, Hunt-Johnson N, Dichter M, True G. Making Connections: Understanding How Screening, Triage, and Referral Processes Can Promote Veteran Housing Stability. Soc Work Public Health. 2019;34: 483–491. doi:10.1080/19371918.2019.1629143

27.	Peretz P, Shapiro A, Santos L, Ogaye K, Deland E, Steel P, et al. Social Determinants of Health Screening and Management: Lessons at a Large, Urban Academic Health System. The Joint Commission Journal on Quality and Patient Safety. 2023;49: 328–332. doi:10.1016/J.JCJQ.2023.04.002

28.	Drake C, Batchelder H, Lian T, Cannady M, Weinberger M, Eisenson H, et al. Implementation of social needs screening in primary care: a qualitative study using the health equity implementation framework. BMC Health Serv Res. 2021;21: 1–16. doi:10.1186/S12913-021-06991-3/TABLES/4

29.	Palakshappa D, Scheerer M, Semelka CTA, Foley KL. Screening for Social Determinants of Health in Free and Charitable Clinics in North Carolina. J Health Care Poor Underserved. 2020;31: 382–397. doi:10.1353/HPU.2020.0029

30.	Montgomery AE, Rahman AKMF, Chhabra M, Cusack MC, True JG. The Importance of Context: Linking Veteran Outpatients Screening Positive for Housing Instability with Responsive Interventions. Adm Policy Ment Health. 2021;48: 23–35. doi:10.1007/S10488-020-01028-Z

31.	Bejan CA, Angiolillo J, Conway D, Nash R, Shirey-Rice JK, Lipworth L, et al. Mining 100 million notes to find homelessness and adverse childhood experiences: 2 case studies of rare and severe social determinants of health in electronic health records. J Am Med Inform Assoc. 2018;25: 61–71. doi:10.1093/JAMIA/OCX059

32.	Biederman DJ, Modarai F, Gamble J, Sloane R, Brown A, Wilson S, et al. Identifying Patients Experiencing Homelessness in an Electronic Health Record and Assessing Qualification for Medical Respite: A Five-Year Retrospective Review. J Health Care Poor Underserved. 2019;30: 297–309. doi:10.1353/HPU.2019.0022

33.	Xie F, Wang S, Viveros L, Rich A, Nguyen HQ, Padilla A, et al. Using natural language processing to identify the status of homelessness and housing instability among serious illness patients from clinical notes in an integrated healthcare system. JAMIA Open. 2023;6. doi:10.1093/JAMIAOPEN/OOAD082

34.	Romanowski B, Ben Abacha A, Fan Y. Extracting social determinants of health from clinical note text with classification and sequence-to-sequence approaches. Journal of the American Medical Informatics Association. 2023;30: 1448–1455. doi:10.1093/JAMIA/OCAD071

35.	Lybarger K, Dobbins NJ, Long R, Singh A, Wedgeworth P, Uzuner Ö, et al. Leveraging natural language processing to augment structured social determinants of health data in the electronic health record. Journal of the American Medical Informatics Association. 2023;30: 1389–1397. doi:10.1093/JAMIA/OCAD073

36.	Guevara M, Chen S, Thomas S, Chaunzwa TL, Franco I, Kann BH, et al. Large language models to identify social determinants of health in electronic health records. npj Digital Medicine 2024 7:1. 2024;7: 1–14. doi:10.1038/s41746-023-00970-0

37.	Hatef E, Chang H-Y, Richards TM, Kitchen C, Budaraju J, Foroughmand I, et al. Development of a Social Risk Score in the Electronic Health Record to Identify Social Needs Among Underserved Populations: Retrospective Study. JMIR Form Res. 2024;8: e54732. doi:10.2196/54732

38.	Chun Y, Roll S, Miller S, Lee H, Larimore S, Grinstein-Weiss M. Racial and Ethnic Disparities in Housing Instability During the COVID-19 Pandemic: the Role of Assets and Income Shocks. Journal of Economics, Race, and Policy. 2023;6: 63–81. doi:10.1007/S41996-022-00109-5/TABLES/5

39.	Publication 28 - Postal Addressing Standards | Postal Explorer. [cited 20 Mar 2024]. Available: https://pe.usps.com/text/pub28/welcome.htm

40.	Bensken WP, Krieger NI, Berg KA, Einstadter D, Dalton JE, Perzynski AT. Health Status and Chronic Disease Burden of the Homeless Population: An Analysis of Two Decades of Multi-Institutional Electronic Medical Records. J Health Care Poor Underserved. 2021;32: 1619. doi:10.1353/HPU.2021.0153

41.	United States Census Bureau. 2023 TIGER/Line Shapefiles. In: The U.S. Department of Commerce [Internet]. 2023. Available: https://www.census.gov/geographies/mapping-files/time-series/geo/tiger-line-file.html

42.	Brokamp C, Beck AF, Goyal NK, Ryan P, Greenberg JM, Hall ES. Material community deprivation and hospital utilization during the first year of life: an urban population-based cohort study. Ann Epidemiol. 2019;30: 37–43. doi:10.1016/J.ANNEPIDEM.2018.11.008

43.	Clinical Classifications Software Refined (CCSR). [cited 14 Apr 2024]. Available: https://hcup-us.ahrq.gov/toolssoftware/ccsr/ccs_refined.jsp

44.	Foraker RE, Rose KM, Whitsel EA, Suchindran CM, Wood JL, Rosamond WD. Neighborhood socioeconomic status, Medicaid coverage and medical management of myocardial infarction: Atherosclerosis risk in communities (ARIC) community surveillance. BMC Public Health. 2010;10: 632. doi:10.1186/1471-2458-10-632

45.	Halpern MT, Bian J, Ward EM, Schrag NM, Chen AY. Insurance status and stage of cancer at diagnosis among women with breast cancer. Cancer. 2007;110: 403–411. doi:10.1002/CNCR.22786

46.	Aubry T, Klodawsky F, Coulombe D. Comparing the Housing Trajectories of Different Classes Within a Diverse Homeless Population. Am J Community Psychol. 2012;49: 142–155. doi:10.1007/S10464-011-9444-Z

47.	Giano Z, Williams A, Hankey C, Merrill R, Lisnic R, Herring A. Forty Years of Research on Predictors of Homelessness. Community Ment Health J. 2020;56: 692–709. doi:10.1007/S10597-019-00530-5/FIGURES/2

48.	Anderson DG, Rayens MK. Factors influencing homelessness in women. Public Health Nurs. 2004;21: 12–23. doi:10.1111/j.1525-1446.2004.21103.x

49.	Chiaramonte D, Clements KAV, López-Zerón G, Ayeni OO, Farero AM, Ma W, et al. Examining contextual influences on the service needs of homeless and unstably housed domestic violence survivors. J Community Psychol. 2022;50: 1831–1853. doi:10.1002/JCOP.22637

50.	Kang S. Severe and persistent housing instability: examining low-income households’ residential mobility trajectories in the United States. Hous Stud. 2023;38: 1615–1641. doi:10.1080/02673037.2021.1982871

51.	Liauw SSL, Mejia-Lancheros C, Lachaud J, O’Campo P, Stergiopoulos V, Hwang SW. Effect of a housing intervention on major cardiovascular events among homeless adults with mental illness. Journal of Social Distress and Homelessness. 2024;33: 263–265. doi:10.1080/10530789.2022.2086670

52.	Lindsay S, Fuentes K, Ragunathan S, Li Y, Ross T. Accessible independent housing for people with disabilities: A scoping review of promising practices, policies and interventions. PLoS One. 2024;19: e0291228. doi:10.1371/JOURNAL.PONE.0291228

53.	Moss AR, Hahn JA, Tulsky JP, Daley CL, Small PM, Hopewell PC. Tuberculosis in the homeless. A prospective study. Am J Respir Crit Care Med. 2000;162: 460–464. doi:10.1164/AJRCCM.162.2.9910055

54.	Robertson MJ, Clark RA, Charlebois ED, Tulsky J, Long HL, Bangsberg DR, et al. HIV seroprevalence among homeless and marginally housed adults in San Francisco. Am J Public Health. 2004;94: 1207–1217. doi:10.2105/AJPH.94.7.1207

55.	Ziff J, Vu T, Dvir D, Riazi F, Toribio W, Oster S, et al. Predictors of hepatitis C treatment outcomes in a harm reduction-focused primary care program in New York City. Harm Reduct J. 2021;18: 1–8. doi:10.1186/S12954-021-00486-4/TABLES/2

56.	Meinbresse M, Brinkley-Rubinstein L, Grassette A, Benson J, Hall C, Hamilton R, et al. Exploring the experiences of violence among individuals who are homeless using a consumer-led approach. Violence Vict. 2014;29: 122–136. doi:10.1891/0886-6708.VV-D-12-00069

57.	Kushel MB, Gupta R, Gee L, Haas JS. Housing Instability and Food Insecurity as Barriers to Health Care Among Low-Income Americans. J Gen Intern Med. 2006;21: 71. doi:10.1111/J.1525-1497.2005.00278.X

58.	Morris T, Manley D, Sabel CE. Residential mobility: Towards progress in mobility health research. Prog Hum Geogr. 2018;42: 112. doi:10.1177/0309132516649454

59.	Bureau UC. Calculating Migration Expectancy Using ACS Data. [cited 17 Jul 2024]. Available: https://www.census.gov/topics/population/migration/guidance/calculating-migration-expectancy.html

60.	Atkins MT, Atkins MT. “On the move, or staying put?” An analysis of intrametropolitan residential mobility and ageing in place. Popul Space Place. 2018;24: e2096. doi:10.1002/PSP.2096

61.	Gillespie BJ, Fokkema T. Life events, social conditions and residential mobility among older adults. Popul Space Place. 2024;30: e2706. doi:10.1002/PSP.2706

62.	Gustavson K, Knudsen AK, Nesvåg R, Knudsen GP, Vollset SE, Reichborn-Kjennerud T. Prevalence and stability of mental disorders among young adults: Findings from a longitudinal study. BMC Psychiatry. 2018;18: 1–15. doi:10.1186/S12888-018-1647-5/TABLES/7

63.	DeLuca S, Wood H, Rosenblatt P. Why Poor Families Move (And Where They Go): Reactive Mobility and Residential Decisions. https://doi.org/101111/cico12386. 2019;18: 556–593. doi:10.1111/CICO.12386

64.	Arundel R, Li A, Baker E, Bentley R. Housing unaffordability and mental health: dynamics across age and tenure. International Journal of Housing Policy. 2024;24: 44–74. doi:10.1080/19491247.2022.2106541

65.	Clarki WAV. Life events and moves under duress: Disruption in the life course and mobility outcomes. Longit Life Course Stud. 2016;7: 218–239. doi:10.14301/LLCS.V7I3.376

66.	Wood GA, Clark WAV, Ong ViforJ R, Smith SJ, Truong NTK. Residential mobility and mental health. SSM Popul Health. 2023;21: 101321. doi:10.1016/J.SSMPH.2022.101321

67.	Hwang SW, Wilkins E, Chambers C, Estrabillo E, Berends J, MacDonald A. Chronic pain among homeless persons: Characteristics, treatment, and barriers to management. BMC Fam Pract. 2011;12: 1–9. doi:10.1186/1471-2296-12-73/FIGURES/2

68.	Vogel M, Frank A, Choi F, Strehlau V, Nikoo N, Nikoo M, et al. Chronic Pain Among Homeless Persons with Mental Illness. Pain Medicine. 2017;18: 2280–2288. doi:10.1093/PM/PNW324

69.	Moss AR, Hahn JA, Tulsky JP, Daley CL, Small PM, Hopewell PC. Tuberculosis in the homeless. A prospective study. Am J Respir Crit Care Med. 2000;162: 460–464. doi:10.1164/AJRCCM.162.2.9910055

70.	Laporte A, Vandentorren S, Détrez MA, Douay C, Le Strat Y, Le Méner E, et al. Prevalence of Mental Disorders and Addictions among Homeless People in the Greater Paris Area, France. Int J Environ Res Public Health. 2018;15. doi:10.3390/IJERPH15020241

71.	Handley CE, Handley CE, Handley CE, Oakley D, Saville CWN. Residential churn moderates the relationship between economic deprivation and psychiatric admission: evidence from Wales. J Epidemiol Community Health (1978). 2020;74: 560–564. doi:10.1136/JECH-2019-213351

72.	Evans M, Chapman A. Residential Immobility and Racial and Ethnic Disparities in Housing Quality. Sociology of Race and Ethnicity. 2024;10: 31–50. doi:10.1177/23326492231207603/FORMAT/EPUB

73.	City of Chicago 2023 Point-in-Time Count & Survey Report of People Experiencing Homelessness.

74.	Lei L, South SJ. Who returned home? The COVID-19 pandemic and young adults’ residential transitions. Adv Life Course Res. 2023;58: 100582. doi:10.1016/J.ALCR.2023.100582

75.	Rowe F, Calafiore A, Arribas-Bel D, Samardzhiev K, Fleischmann M. Urban exodus? Understanding human mobility in Britain during the COVID-19 pandemic using Meta-Facebook data. Popul Space Place. 2023;29: e2637. doi:10.1002/PSP.2637

76.	Frederick TJ, Chwalek M, Hughes J, Karabanow J, Kidd S, Aube C, et al. HOW STABLE IS STABLE? DEFINING AND MEASURING HOUSING STABILITY. J Community Psychol. 2014;42: 964–979. doi:10.1002/JCOP.21665

77.	Phillips DC. Measuring Housing Stability With Consumer Reference Data. Demography. 2020;57: 1323–1344. doi:10.1007/S13524-020-00893-5/FIGURES/8

78.	Clark A. The role of residential mobility in reproducing socioeconomic stratification during the transition to adulthood. Demogr Res. 38. doi:10.4054/DemRes.2018.38.7

79.	Coulton C, Theodos B, Turner MA. Cityscape Residential Mobility and Neighborhood Change: Real Neighborhoods Under the Microscope. Cityscape: A Journal of Policy Development and Research •. 2012;14.

**Supporting information**

**S1 Table. Top ten diagnoses and prevalence per address churn group.**
